# Towards an Age-Phenome Knowledge-base

**DOI:** 10.1186/1471-2105-12-229

**Published:** 2011-06-08

**Authors:** Nophar Geifman, Eitan Rubin

**Affiliations:** 1 Shraga Segal Department of Microbiology and Immunology, Faculty of Health Sciences and The National Institute for Biotechnology in the Negev, Ben Gurion University, Building 39, Israel

## Abstract

**Background:**

Currently, data about age-phenotype associations are not systematically organized and cannot be studied methodically. Searching for scientific articles describing phenotypic changes reported as occurring at a given age is not possible for most ages.

**Results:**

Here we present the Age-Phenome Knowledge-base (APK), in which knowledge about age-related phenotypic patterns and events can be modeled and stored for retrieval. The APK contains evidence connecting specific ages or age groups with phenotypes, such as disease and clinical traits. Using a simple text mining tool developed for this purpose, we extracted instances of age-phenotype associations from journal abstracts related to non-insulin-dependent Diabetes Mellitus. In addition, links between age and phenotype were extracted from clinical data obtained from the NHANES III survey. The knowledge stored in the APK is made available for the relevant research community in the form of 'Age-Cards', each card holds the collection of all the information stored in the APK about a particular age. These Age-Cards are presented in a wiki, allowing community review, amendment and contribution of additional information. In addition to the wiki interaction, complex searches can also be conducted which require the user to have some knowledge of database query construction.

**Conclusions:**

The combination of a knowledge model based repository with community participation in the evolution and refinement of the knowledge-base makes the APK a useful and valuable environment for collecting and curating existing knowledge of the connections between age and phenotypes.

## Background

The relationship between age and human health has been extensively investigated over the years and age has been linked to a plethora of so-called age-related diseases [1]. A patient's age may effect the course and progression of a disease [2,3] or may be an important factor in determining the right course of treatment [4]. As a result of these endeavors, a significant quantity of data exists linking specific ages or age ranges with disease, as well as with other clinical phenotypes such as 'normal' parameter values from blood tests.

Currently, data relating to age-phenotype associations are not systematically organized and cannot be studied methodically. For example, searching for scientific articles describing the phenotypic changes reported to occur at a given age is not possible for most ages. Consider for example, the search for data on the change in serum hemoglobin levels in post-partum women aged 30. It is fairly straightforward to discover works that discuss postpartum hemoglobin level but simply searching for works associating hemoglobin with the term 'age 30' yields a huge volume of work that mentions either the age of the individual or the average age in the cases that were studied. It is then necessary to find, by reading through the papers or abstracts, that subset of these works that discuss some events or trends relating to a particular age. For age-related phenotypic changes to be useful for medical research, not only are interactive searches much needed, but the information retrieved should be amenable to computational analysis. One approach to achieving these goals is by means of a knowledge-base in which age-related events, trends and phenotypic changes are recorded in a structured knowledge representation model. With such a knowledge-base, it should be possible to support dynamic queries about events known to occur at specific ages or within age ranges. Moreover, such a knowledge-base will allow mining of existing knowledge for novel insights into age-related processes.

Over the years, many efforts have been made to represent biomedical knowledge and make it accessible for structured querying and computational mining, leading to the emergence of many bio-medical knowledge-bases [5]. However, most of the knowledge-bases that are concerned with human phenotypes do not represent the connection of these phenotypes to age. One such knowledge-base is the Human Phenotype Ontology (HPO) [6], which provides a standardized vocabulary of phenotypic abnormalities encountered in human disease. The HPO includes a set of terms which allow the description of some age-related information such as age of onset (for example, the term 'Onset in early adulthood') but detailed knowledge linking age to phenotype is not included. Online Mendelian Inheritance in Man (OMIM) [7], another major phenotype-related knowledge-base, maintains knowledge about age and the links to disease, yet this knowledge is not structured and includes only a limited range of phenotypes, such as genetic disorders.

We present here the Age-Phenome Knowledge-base (APK), in which knowledge about age-related phenotypic patterns and events is modeled and stored. The knowledge-base holds a structured representation of knowledge, derived from the literature, about clinically-relevant traits and trends which occur at different ages such as disease symptoms and disease propensity. In addition, the database underpinning the knowledge-base can hold information about trends and trend changes in biomarker values and anthropomorphic properties. Disease and age are described using ontologies, allowing abstraction in searches (for example, searching for evidence linking "infectious diseases" and "children" instead of searching for a specified list of diseases and a range of ages as would be required for a relational database search). The knowledge-base is populated, using computational text-mining tools and human curation, with age-related knowledge as found in the medical and scientific literature. In addition, the knowledge-base contains patterns extracted from raw clinical data. Earlier analysis of such patterns, using data sources such as the National Health and Environmental Nutrition Survey and the Soroka Maternity Database showed that non-trivial age trends can be extracted from raw clinical data [8,9]. By combining these patterns with the age-related knowledge to be found in the research corpus, it should be possible to suggest new ways to consider patient age in the improvement of patient care and to propose new research avenues.

## Results

### Overall Structure of the APK

**Figure 1:**
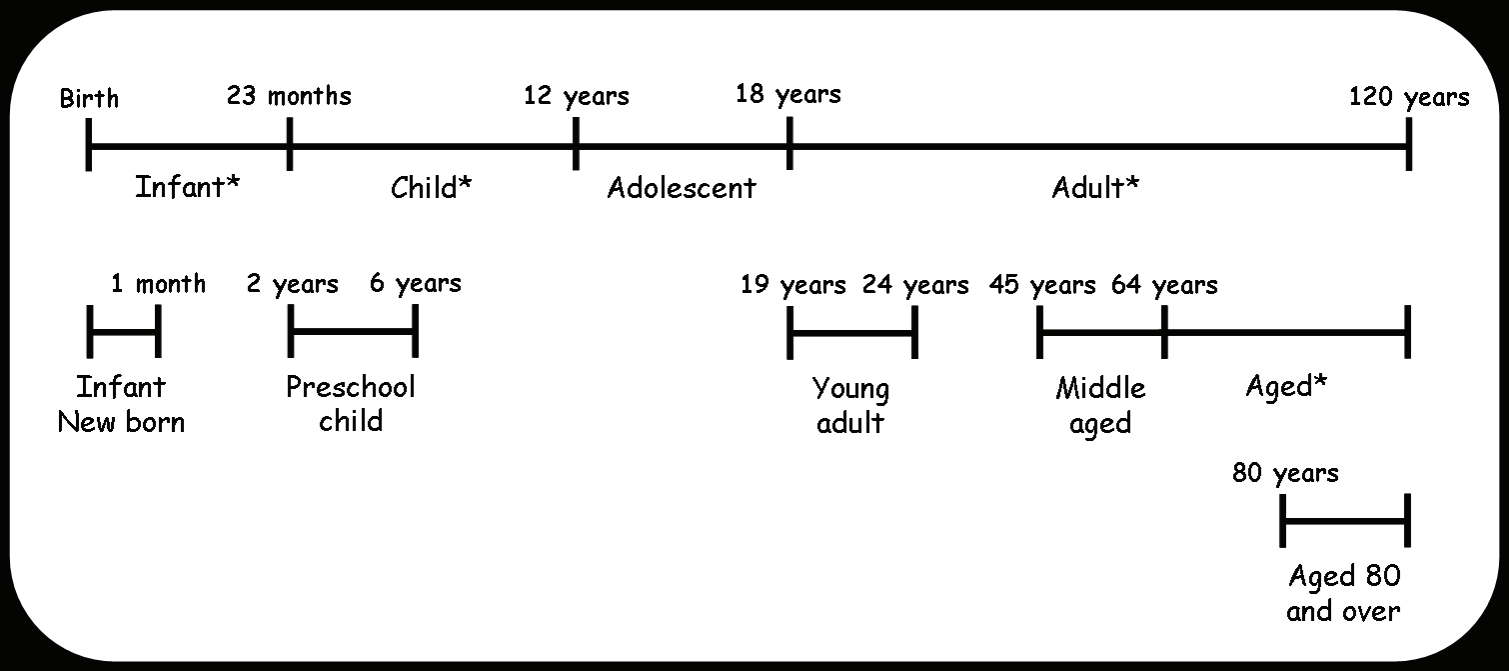
**Age-range classes defined by the Age Ontology**. Age-range classes were generally defined based on the MeSH age range definitions, with minor changes introduced to improve consistency. In the Age Ontology, age-range classes that are contained within another age-range class are defined as subclasses of that class. Each age-range class is made disjoint from all other age-range classes since each specific age can belong to only one age range class. * This age range was modified from the MeSH definition.

The Age-Phenome Knowledge-base contains evidence connecting specific ages or age groups to phenotypes, such as clinical traits. It comprises a relational database, ontologies and a wiki used for knowledge representation to the user community. The database stores all the evidence instances and the ages and phenotypes to which they are linked. Age and diseases are described using the Age Ontology and the Human Disease Ontology respectively. The Age Ontology is a very simple ontology, developed specifically for this research, that allows ages to be represented by grouping them into classes similar to those defined in the Medical Subject Headings (MeSH) controlled vocabulary [10]. In the Age Ontology, age is defined as the time which has passed since birth and is an attribute of a person (Figure 1). Minor changes were introduced to the MeSH age definitions in order to improve their formal logic consistency. For example, in MeSH the term 'Child' is defined as 'A person 6-12 years of age' while the term 'Preschool child' is defined as 'A person 2-5 years of age'. To make the class 'Preschool child' logically a sub-class of 'Child' in the Age Ontology, the definition of 'Child' had to be changed to 'A person 2-12 years of age'. Though the Age Ontology currently is small and has a very simple design, its hierarchal structure is serving the intended uses of the APK. In order to achieve a richer description of ages this ontology will be further developed in the future.

The second ontology in use for the APK is the Human Disease Ontology (DO) [11] which organizes and describes diseases and was designed for use in clinical research and in Medicine. DO was incorporated into the APK to represent diseases as one form of phenotype. An additional resource, a collection of clinical traits not found in DO but occurring in the APK source documents, allows the scope of the APK to be extended to cover other clinical age-related processes, such as changes in common clinical markers. This collection may be improved by adding higher-order terms that abstract its terms, giving a meaningful structure to the collection in the context of the APK. This kind of abstraction could be supported by importing relevant terms from the SNOMED-CT clinical terminology [12]. However, since this ontology requires licensing, its incorporation into the APK will be considered for a future enhancement of the APK.

Each evidential text fragment stored in the knowledge-base is linked to the specific age (or age range) and phenotype(s) mentioned by it. Five different types of relationships were used to describe the links between evidence and age. These five types describe the most commonly occurring age-phenotype relationship contexts: 'Age of Onset', 'Age of Diagnosis', 'Age of Observation', 'Age of Occurrence' and 'Age of Evaluation'. 'Age of Onset' is used to link age to evidence describing the age of the beginning of manifestation of the phenotype. 'Age of Diagnosis' is used when the evidence describes the age at which a phenotype (usually a disease) is first diagnosed. 'Age of Observation' is used in cases where an observation is made at a specific age, for example a change in the phenotype 'hemoglobin level'. 'Age of Evaluation' is used when the evidence describes an evaluation of a phenotype in a certain age or age range, only indirectly asserting that these ages may have been selected to evaluate the phenotype for a reason. 'Age of Occurrence' is used to link age to evidence when the evidence describes a non-dependent occurrence of phenotype at a certain age but without asserting that the age has a direct effect on the phenotype.

**Figure 2:**
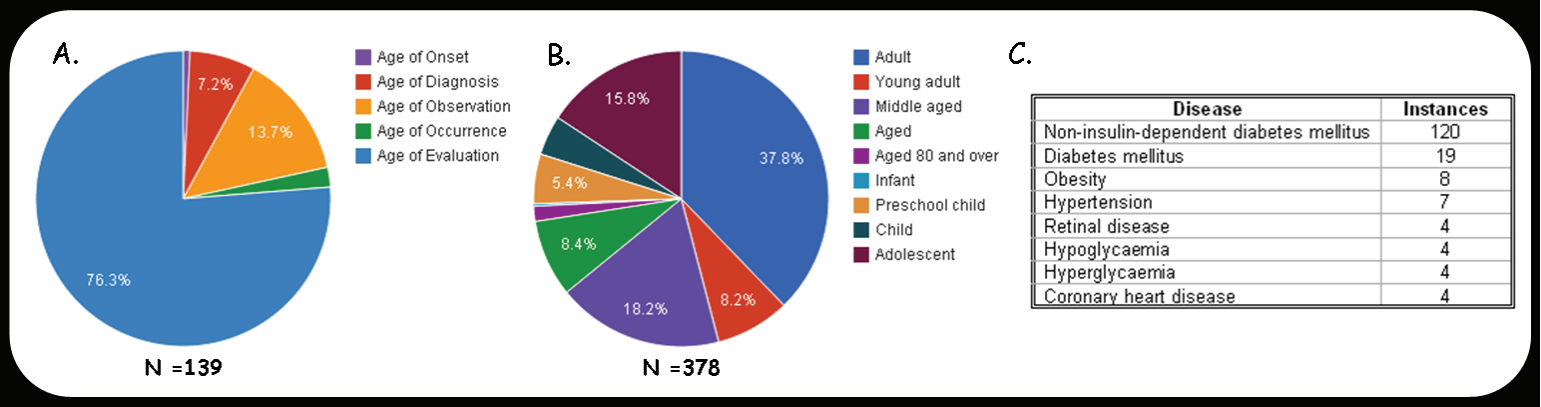
**The Age-Phenome database's contents summary**. A. Instances per relationship type. Only instances extracted from PubMed abstracts are shown. B. Instances per Age Ontology age class. An instance is assigned the most specific Age Ontology class which contains the whole of the age range that that instance linked to. C. The top diseases from DO linked to instances in APK.

To demonstrate the approach used here for capturing and making medical knowledge linking age to disease available, the APK was populated with concrete examples. Using a simple text mining tool developed for this purpose, 139 instances of age-phenotype associations were extracted from 100 abstracts related to non-insulin-dependent Diabetes Mellitus. A further 239 examples of links between age and phenotype were extracted from clinical data obtained from the NHANES III survey. The knowledge-base was then populated with the resulting 378 evidence instances, together with their mapping to the corresponding age and phenotype. A summary of the data stored in the APK is provided in Figure 2. During the manual population of the knowledge-base, it was noticed that it is sometimes challenging even for a human reader to select the relationship type that best describes the type of age referred to in the text segments. For example, in the text "*Serum and lipoprotein lipids were examined in 133 newly diagnosed (type II) diabetic patients (70 men, 63 women), aged 45-64 yr, and in 144 randomly selected non-diabetic control subjects of similar age (62 men, 82 women). The serum total cholesterol levels in diabetic and non-diabetic subjects were similar, but the HDL-cholesterol levels were lower and the serum total triglyceride levels higher in the diabetic than in non-diabetic subjects*" there are two statements made regarding the age range 45-64 years. The first is that the patients investigated were 45-64 years old when diagnosed with Diabetes, and the second is that this age range was used when evaluating cholesterol and triglyceride levels in diabetic patients. In order to capture the age-related knowledge in this text, two evidence instances were created. The first (APKID_138) linking Diabetes to the ages 45-64 with the 'Age of Diagnosis' relationship; and the second (APKID_101) linking these ages to 'Diabetes', 'serum total cholesterol', 'HDL-cholesterol levels', and 'serum total triglyceride levels' with the 'Age Of Evaluation' relationship. Automatically resolving this kind of relationship assignment would require sophisticated natural language processing tools and it seems unlikely that a fully automatic system could be developed in the near future. It was therefore decided to harness the "wisdom of the crowd" by engaging user communities in identifying and correcting erroneous machine interpretations of text. This approach is manifested in the development of "age cards" that were made available to interested readers as pages in a wiki. The wiki technology was successful in engaging a community of relevant readers in amending and extending the knowledge items, with Wikipedia as the shining example [13]. As in Wikipedia, readers of the age-phenome Age Cards can add comments, propose new entries and correct or delete existing entries. For the development of the APK, the community edits are collected from the history section of the Wiki, and incorporated into the knowledge-base following manual curation.

Many scientific texts refer to age ranges, such as 2-7 years, rather than one specific age. In such cases, the evidence instance is linked to the age range defined in the database by the minimum and the maximum values of the range. In some cases, the age range is not fully constrained, for example "30 years or less" and there are two possible approaches to resolving this issue. The evidence instance could be linked only to the age specifically mentioned, that is, 30 years in this example. In this case, some information will most likely be missed. The second option, and the one employed in the APK, is to link the evidence instance to all the possible ages covered by the expression, producing links to 0-30 for "30 years or less". Although this approach might add erroneous links, since the author might not have intended all ages under 30 years, no information will be missed. In future development of the APK, more sophisticated natural language processing will be used to attempt to locate and extract a more precise age range from the full article since this is likely to appear in the methods or results sections. For example, in a text discussing "children", these sections may contain the exact range such as 3-6 years of age.

### Using the APK

**Figure 3:**
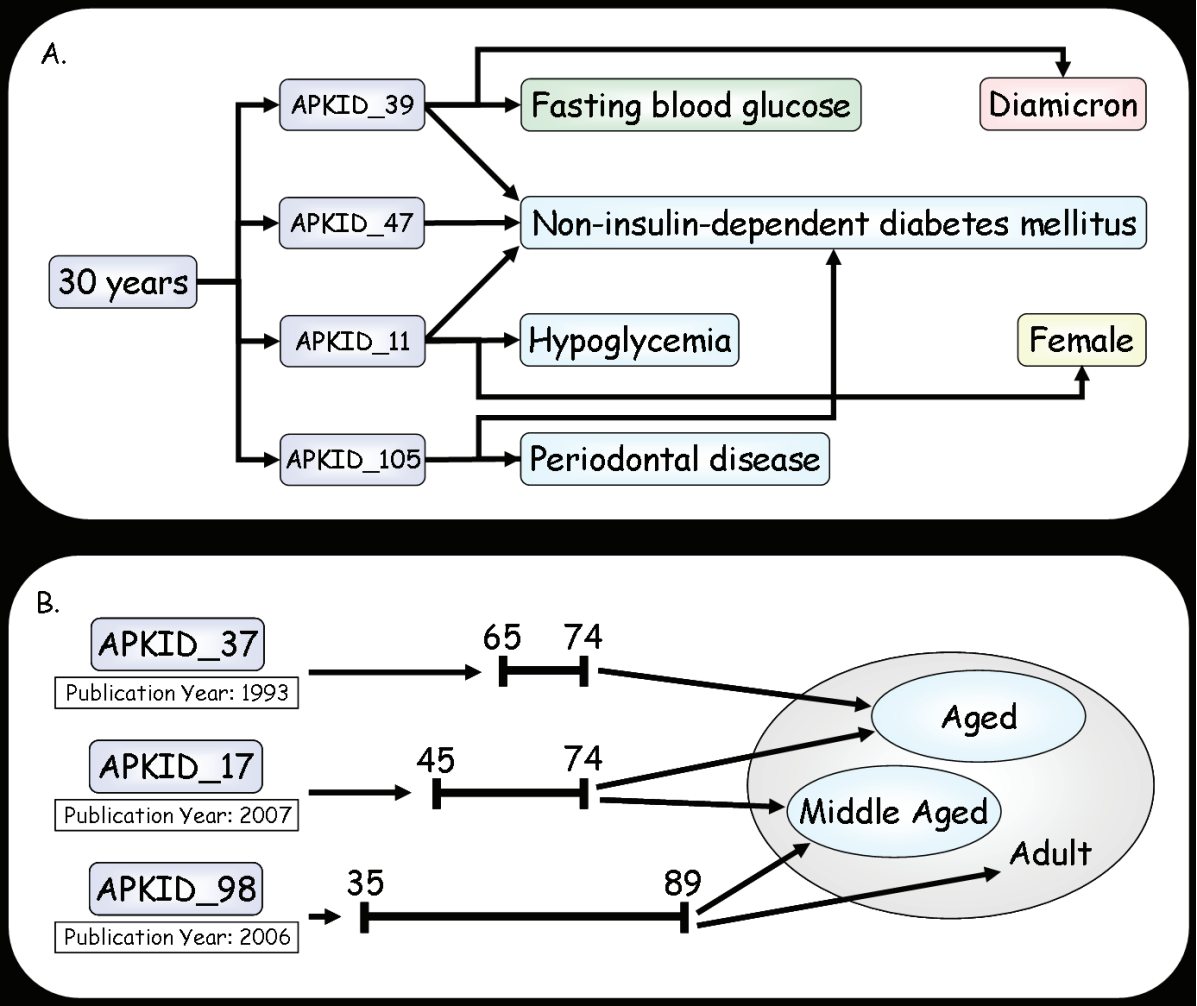
**Querying the APK**. A. the APK was queried for phenotypes linked to age 30 years. The results show 4 (of 19) evidence instances that were found linked to this age via the 'Age Of Evaluation' relationship and the different phenotypes linked to these evidence instances. B. the APK was queried for ages linked to the phenotypes 'Non-insulin-dependent Diabetes Mellitus' and 'Hypertension'. The query was restricted for evidence instances with a publication year after 1990 and the age-range classes to which the resulting ages belong to were also extracted from the Age Ontology. The results of this query show three evidence instances linked to both phenotypes, their publication year, the ages each evidence instance is linked to and the age-range classes they belong to.

One way to use the APK is to extract all the information linked via evidence instances to a specific age or age-range. This can answer questions such as "what type of studies involve 30 year old individuals?" In the example shown in Figure 3A, the age '30 years' was queried and the phenotypes linked to this age via the 'Age of Evaluation' relationship are shown.

Another way to use the APK is to locate all the specific ages and the age-ranges in the Age Ontology that are linked (via evidence instances) to a specific phenotype of interest or to a combination of phenotypes. In the example shown in Figure 3B, all the ages linked to 'Non-insulin-dependent Diabetes Mellitus' AND 'Hypertension' were extracted, with the results being filtered by their year of publication. From the query results, it may be seen that in the APK there are three evidence instances in which these two diseases (phenotypes) were evaluated and that the age-ranges used for the evaluations partially overlap.

It must be stressed that the examples provided here are somewhat simplistic. More complex queries can use, for example, the Age Ontology and the Disease Ontology's hierarchal structures. It is possible, for example, to search for evidence linking childhood infectious diseases and Diabetes, using the Age Ontology to translate "childhood" into an age range and to enumerate those diseases in the APK that are associated with the "infectious diseases" class in the DO. Thus, as the APK becomes more extensively populated with evidential instances, queries can become more complex and their results are expected to become more interesting and informative.

It should be noted that, in the current implementation, conducting complex queries in the APK requires some familiarity with the SQL language.

### The Age-Phenome Wiki

The Age-Phenome Wiki was developed as a means to share the knowledge stored in the APK and to harness the knowledge and expertise of the user community in further developing the APK. This Wiki is constructed of 'Age Cards' which are generated in a semi-automatic fashion. For each specific age there is an Age-Card which contains all information linked to that age to be found in the APK. Each Age-Card has two versions. The first version is for viewing the information and uses highlighted text and colors for clearer presentation. The second version is created for community edits, avoiding formatting instructions (such as coloring) making it easy for untrained users to edit the text with a simple editor. Edits are integrated back into the database, allowing the knowledge-base to grow and become more accurate based on readers' input. Currently, this integration is carried out manually by curators who review the revision history of the cards. However, as the volume of editing increases, the tools that are currently used to read and parse the literature will be used to parse the edits, reducing the manual review and editing load.

## Discussion

There is a wide consensus that age is an important factor when considering phenotypic changes in health and disease. Though relationships and dependencies between age and phenotype can be extracted from clinical data and are widely discussed in medical literature, there is at present no system which allows methodical investigation of the biological meaning of such relationships. Even the task of assembling all the information about a specific age is labor intensive, requiring the review of massive bodies of literature, and may only yield a small number of relevant works.

The Age-Phenome Knowledge-base is being developed in order to create a knowledge representation of multiple types of links between age and phenotypes. These links are supported by various forms of evidence, such as excerpts from scientific publications and results of clinical data analysis. Once the knowledge-base is richly populated with both evidence instances and with relationships which connect age to phenotype via the evidence instances, it can be used to answer questions regarding different ages and how they might affect phenotype (and vice versa). Several potential uses may be envisaged for the APK, the most straightforward of these being to present age-phenotype interrelationships in a readable and searchable form. To reach its critical mass of current knowledge, it seems very likely that the development of the APK will require a community effort, much like other knowledge-bases [14]. To facilitate this effort, a wiki-structured representation of the knowledge-base has been implemented. Through this wiki, users are able to review and amend the information stored in the knowledge-base, flagging erroneous interpretation of the text or suggesting alternative or contradictory evidence. For example, if an outdated abstract connects a disease to a specific age and this connection has since been disproved then the user can edit the entry, suggesting the correction to the curators. To facilitate the inclusion of user-added knowledge whilst protecting the integrity of the knowledge-base, user suggestions are processed (manually or automatically) prior to their transfer back into the knowledge-base.

The principal use of the knowledge-base is to answer questions regarding linkages between age and phenotype. An example of this kind of use is the observation made in our lab, that post-partum hemoglobin peaks at the age of 30. This raises the question of what other phenotypic changes occur at that age. With the APK, it is possible to query the knowledge-base and find which phenotypes are linked to this precise age (30), or approximate ages (29-31, for example) or the same age group (adults). The query results can be filtered by types of evidence which link the age and phenotype and by parameters such as publication dates of text evidence. Also, since phenotypes are defined with an ontological structure, the query could be restricted to phenotypes that pertain to a specific physiological system. The APK could also be used to assist users in experimental design by identifying the optimal age or age group for investigating a specific phenotype.

The use of formal ontologies for representation of phenotypes and age allows queries at any level of abstraction-generalization that interests the user. Phenotypes linked to a certain age can be viewed at different resolutions (high level or detailed) and ages linked to a specific phenotype can be viewed both as specific ages such as 30 years old or more general age-range classes, like adult. This approach can be further extended to consider unsupervised searches for patterns. Given a well populated database, it should be possible to generate promising hypotheses by detecting linkages between phenotypes and recurrent critical ages.

Another use of the knowledge-base is to redefine meaningful age groups. Currently, age groups in the Age Ontology are fashioned after the definitions used in MeSH [10] and seem to be rather simplistic. Once the knowledge-base is more extensively populated with evidence instances, it should be possible to re-evaluate these classifications of ages. For example, if many evidence instances indicate some age-ranges that are not currently defined as a group, this may indicate that the age-range is important with respect to a specific aspect of Medicine or Biology and should be added to the Age Ontology.

### Future development of the APK

The APK is currently at an early stage of development and further work is needed for the knowledge-base and wiki to reach their full potential. One of the goals of this paper is to engage potential users in contributing to the APK, either by editing the relevant wiki entries or by directing attention (also through the wiki) to specific areas in which age-related knowledge is likely to be helpful. Evaluation of user feedback will also allow the improvement of the Age-Phenome Wiki.

Presently, adding knowledge to the APK requires manual editing by domain experts. In order for the APK to become widely used, it must be extended to include far more complete descriptions of existing knowledge. It is worth noting here that most of the applications of the APK involve inspection of the results by domain experts. As a result, a process that adds knowledge automatically to the APK, even with only moderate accuracy, could still be very useful. Consequently, tools are now being developed that will be able to extract relevant knowledge linking ages to phenotypes from the millions of processed PubMed abstracts. A more robust knowledge extraction pipeline is being developed that will mine abstracts listed in PubMed for relevance to any age, and find any of the predefined relationships to phenotype, such as different diseases. For this next stage, existing information extraction techniques and programs [15,16] will be used with their possible adaptation to this specific use.

From the experience gained in this work, it has become evident that it is necessary to analyze the full text of the relevant literature; some abstracts are ambiguous and some lack critical information. It is therefore planned to mine Pub Med Central [17] to seek age-phenotype relationships in whole papers. In the future, collaborations with relevant publishers and content providers may allow even more complete coverage of the literature. Advancing to analysis of the full text of the relevant papers will require more powerful text mining techniques, and will involve specific adjustment of the pipeline for complete text analysis, for example treating the methods and the results sections differently.

## Conclusions

The design, development and preliminary testing of a knowledge-base of age-related phenotypic changes in humans are described. At the current level of knowledge represented in the APK, it is limited to being a demonstration of the concept and its potential uses. Nevertheless, the Age-Phenome wiki may engage the research community and bring about rapid population of the knowledge-base with knowledge from specific domains such as Diabetes. The APK will immediately make this knowledge searchable in ways which are not currently achievable in other knowledge sources. The combination of a structured knowledge model along with community participation in its evolution, makes the APK a promising platform for collecting existing knowledge concerning the interplay between age and phenotypes.

## Methods

### The Age-Phenome database

**Figure 4:**
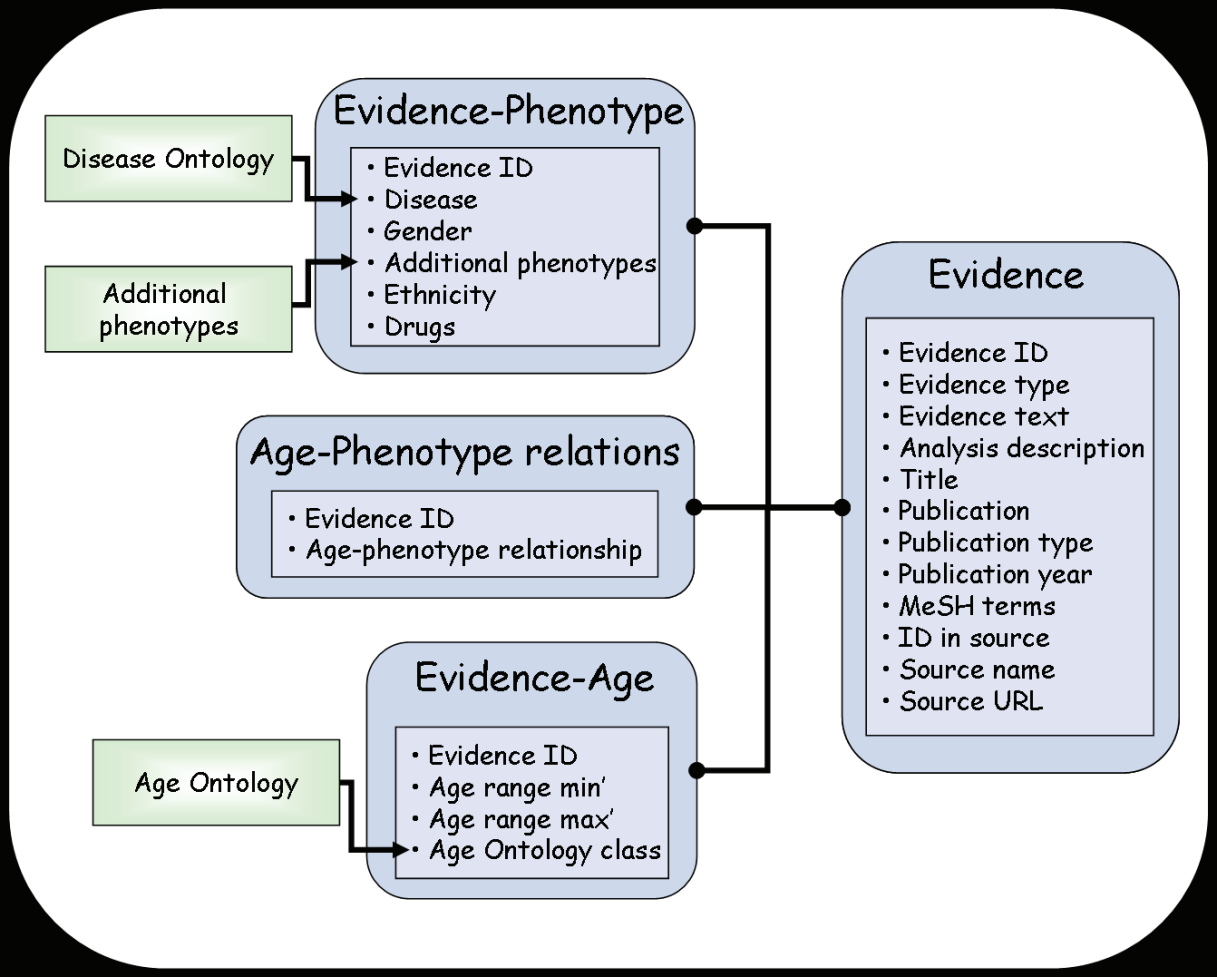
**The Age-Phenome Database's overall structure**. The Age-Phenome database comprises of 4 main data-tables: (1) Evidence, (2) Evidence-age, (3) Evidence-phenotype, and (4) Age-Phenotype relationships. In the Evidence table each evidence instance has a unique identifier and is characterized by: 'Evidence type', 'Evidence text', 'Analysis description' (for evidence computationally derived from clinical data rather than literature), 'Title', 'Publication', 'Publication type', 'Publication year', 'MeSH terms' (for evidence taken from literature), 'ID in source', 'Source name' and 'Source URL'. The 'Evidence text' field holds an evidential text fragment. In the evidence-age table, each entry records the age or age range that the evidence instance is linked to, using 'range min' and 'range max' fields to describe ranges as well as single ages (using identical range min and max values). In the evidence-phenotype table, values for the 'disease' and 'Additional phenotypes' fields are taken from the Disease Ontology, and a locally compiled additional phenotypes list (phenotypes such as different blood, available as Additional file 4), respectively. In the age-phenotype-relationship table, each evidence instance is linked to one of five different relationships ('Age of Onset', 'Age of Diagnosis', 'Age of Occurrence', 'Age of Observation' and 'Age of Evaluation').

The database, written in MySQL [18], comprises four main data tables (Figure 4):

(i) The evidence table contains all the pieces of evidence and their description.

(ii) The evidence-age table contains a description of the age information found in each evidence instance.

(iii) The evidence-phenotype table links phenotypes to each evidence instance.

(iv) The age-phenotype-relationship table is used to record the type of relationship linking the age and phenotype mentioned in each evidence instance.

### Ontologies

The Age Ontology was developed and maintained using the Protégé 4.0 Beta OWL editor [19], and was written in the OWL-DL language. The ontology is available as Additional file 1. The Human Disease Ontology (DO) [11] was downloaded (December 2009) from the Open Biomedical Ontologies Foundry [20] and imported into the knowledge-base.

### Information extraction

As a test case, PubMed [17] was searched for articles on non-insulin dependent Diabetes Mellitus (Type 2 Diabetes). The search was restricted to human-related articles with all the 'ages' search options selected in the 'Advanced' search settings, as of November 2009. This search yielded approximately 22,000 abstracts.

A script, implemented in Perl, used a list of regular expressions (available as Additional file 2) to determine which of the abstracts were related to age. One hundred of the resulting abstracts were then manually searched for the specific age being addressed, the type of connection to phenotype and the specific disease. From these abstracts 139 instances for the knowledge-base were created, each describing the link of a specific age (or age range) to a disease or set of diseases. Basic age-related information contained in these abstracts was captured for each instance in the APK.

### Clinical data

Data obtained from the NHANES III survey [21] was analyzed for age related trends. For a full description of the analysis see the "A method for identifying critical ages from clinical laboratory cross-sectional data" published at: http://rubinlab.med.ad.bgu.ac.il/APK_clinical.html.

An evidence instance was generated for each observation made from the data analysis; 239 evidence instances were added to the knowledge-base, together with their connections to age and phenotype.

### The Age-Phenome Wiki

The Age-Phenome Wiki was built using the MediaWiki platform [22] and is available at: http://age-phenome-wiki.med.ad.bgu.ac.il

### Database availability

The APK database is available as a MySQL dump in Additional file 3 and a list of additional phenotypes that were mapped to evidence texts in APK can be found in Additional file 4.

## Authors' contributions

NG and ER conceived the idea, designed the research and wrote the article; NG conducted the research. All authors have read and approved the final manuscript.

## Supplementary Material

Additional file 1 **The Age Ontology**. This file contains the Age Ontology, developed in the OWL-DL language. Click here for file

Additional file 2 **Age relating terms**. This file contains a list of regular expressions that were used to determine which of the abstracts were related to age. Click here for file

Additional file 3 **The APK database**. This compressed folder contains the APK database as a MySQL dump. Click here for file

Additional file 4 **List of additional phenotypes**. This file contains a list of additional phenotypes that were mapped to evidence texts in APK. Click here for file
